# Notes upon the Diagnosis of Angina Pectoris

**Published:** 1910-01

**Authors:** Glentworth R. Butler

**Affiliations:** Brooklyn, N. Y.; Visiting Physician, Methodist Episcopal and Brooklyn Hospitals; Consulting Physician, Bushwick Central Hospital


### Notes Upon the Diagnosis of Angina Pectoris

By GLENTWORTH R. BUTLER, M. D., Brooklyn, N. Y.
Visiting Physician, Methodist Episcopal and Brooklyn Hospitals; Consulting Physician, Bushwick Central Hospital.

[*The Archives of Diagnosis*, October, 1909.]

ANGINA pectoris is a symptom and not an entity. But its clinical manifestations are so striking and its import so grave that it occupies a recognised place on the nosological list.

Pathology.—The anatomical pathology of angina pectoris is fairly uniform, although it may occur in connection with a number of abnormal conditions of the heart and arteries. But almost invariably it is associated with sclerotic changes affecting either the main divisions of the coronary arteries, or the root of the aorta with more or less stenosis of the openings of the coronaries. Narrowing of the lumen of the coronary arteries, with a consequent impairment of the supply of blood to the heart muscle, constitutes the anatomical condition which is apparently essential to the production of a true angina pectoris.

The physiological pathology of stenocardia is not definitely settled. Marked or extreme sclerosis of the coronary arteries frequently exists without giving rise to angina pectoris. While ligation of both coronaries will be followed by slowing and irregularity of the heart-beat, with consequent dilatation, and death within a few moments, there is seldom any evidence of pain. Moreover, in the clinical instances of sudden death due to thrombosis or embolism of the coronaries, pain is by no means a common symptom. It is evident that stenosis of the coronaries is but one of the necessary conditions, and is not, by itself, sufficient to cause breast-pang. The role played by coronary stenosis in the production of angina is strikingly analogous to the part taken by disease of the arteries of the lower extremities in the causation of intermittent claudication. Thus when there has been thrombosis of the iliacs, or advanced sclerosis affects the arteries of the legs, the supply of blood to the muscles may be quite adequate when they are at rest. But after brisk walking the blood does not flow in sufficient quantity to the muscles, and as a result of a relative ischemia, pain, spasm, and transient loss of power in the muscles will occur—the "painful limping" of some writers. After resting for a time the pain and weakness disappear.

The theory of a transient ischemia of the heart-muscle, due to coronary stenosis, possibly also to angiospasm affecting these arteries, goes far in explaining the paroxysms of angina pectoris. No one, however, believes that this explanation is complete and wholly satisfactory, mainly because of the fact that coronary stenosis is of common, and angina pectoris of rather rare, occurrence. It is evident that there are other causes, at present mainly conjectural, which are instrumental in the genesis of the disease.

It is quite probable that an increased degree of irritability of the nervous apparatus of the heart must be predicated. Still more probable is it that some disturbance of one or more of the various inherent properties of the heart-muscle itself is to be taken into account. Since the work of Mackenzie, Erlanger, and others, the myogenic theory of heart activity can be regarded as resting upon a fairly firm foundation. According to this theory the heart-muscle exhibits five inherent and fundamental properties, each distinct from the other, and each capable of varying, quite independently, in its degree of activity. These properties are, *first*, the power of generating automatic rhythmic stimuli, which, under normal circumstances, originate in the venous openings of the right heart, and pass downward to the ventricles, via the muscle-bundle of His, by virtue of the *second* property of the cardiac muscle, its conductivity. Such stimuli do not pass by way of the nerves. By the discovery of these facts, the cardiac nervous apparatus has been relegated to a place of minor importance. Stimuli may arise at other points than those in the right auricle, but, normally, do not affect the rhythm of the heart. In certain cases of disease the abnormal stimuli may overcome the normal, even to the extent of reversing the usual direction of conduction. The remaining three properties of the cardiac musculature are those common to all muscular tissues, namely, irritability, contractility, and tonicity.

These inherent properties of the heart-muscle may be modified, independently one of the other. Thus the presence of certain salts seems necessary for the production of the rhythmic stimuli. The circulating blood may contain poisons, either organic or inorganic, which will increase or depress the functions of the heart-muscle. The cardiac nervous apparatus, although it does not originate the movements of the heart, has the power of regulating them. Stimulation of the pneumogastric depresses, of the accelerator nerve increases, the production of rhythmic stimuli, the rate of conduction, and the contractility of the heart muscle, with corresponding effects upon the heart-action.

Recalling these functions of the heart-muscle, and the fact that coronary disease is practically synonymous with degenerative myocardial changes, it is obvious that such changes are instrumental in producing at least some of the phenomena observed in true stenocardia. It is probable that disease of certain areas of the heart-muscle predisposes more definitely to the occurrence of angina than disease of other portions. This difference may be due in part to the presence of a richer or more important nerve supply in the different areas; in part to the presence or absence of more or less free anastomoses between the finer branches of the coronary arteries.

There remains a question as to the part taken by the vasomotor nervous system in the anginal paroxysm. A varying importance has been attached to the element of angiospasm affecting the coronaries, the relative ischemia thus produced giving rise to the same symptoms as those due to the intermittent ischemia of organic stenosis. That coronary spasm is a factor in the paroxysm can scarcely be doubted, especially, perhaps only, in the early stages of the disease, before the coronaries have undergone such a degree of thickening or calcification as to render them incapable of response to vasomotor impulses. The probability of this statement is borne out by the vasomotor phenomena which, to a diverse extent, accompany a considerable proportion of cases of the disease. These are: coldness and numbness of the extremities, pallor, sweating, faintness, increase of arterial pressure, and the fact that attacks are often precipitated by strong emotions. That false angina pectoris—cardiac pain without discoverable disease of the coronaries—is due to disturbed innervation can hardly be questioned. It is classed, with but few reservations, as a cardiac neurosis.

I can testify to the existence of angiospasm in at least one instance of true angina. The patient presented indubitable evidences of arteriosclerosis, and had been subject for two years to recurring severe precordial pain on exertion or excitement. The maximum arterial pressure had just been determined to be 245 mm. At this moment the patient complained of a beginning attack of breast pain. Within two minutes the pressure had risen to 290 mm. The attack was accompanied by pallor of the face, and coldness and numbness of the hands. Nitroglycerin promptly relieved the pain, with a reduction of the pressure to 240 mm.

Varieties and Course.—Angina pectoris varies greatly in its mode of onset and its severity. Death may take place during or immediately after the first seizure; or there may be recurrences every few hours for several successive days, the heart growing weaker and finally stopping, or the attacks may be distributed over a number of years. Frequently death occurs, not during a paroxysm, but suddenly and without an immediate warning.

Diagnosis.—The diagnosis of true angina is, in the greater number of instances, readily made. But there are some pitfalls into which a careless foot may lead us. Of these one must beware of the unusual localisations of anginal pain. In five out of thirty-two true anginas seen within the past ten years, the pain began, and was most severe, in the left wrist; in three, in the back of the neck and the shoulders; in two, in the epigastrium; in two, in the mid-abdomen. In such instances precordial pain is present, but may be relatively so slight that it is often minimised by the patient. It is important to regard with suspicion, as of possible anginal origin, all sudden painful gastric or intestinal disturbances of brief duration, accompanied by eructations or flatulence, when occurring in middle-aged adults, especially men. Such an event may either be the exciting cause of an anginal paroxysm, or, I believe, form an integral part of, or, alone, constitute a seizure.

For clinical convenience patients with angina may roughly be divided into two classes according to the mode of onset and the course of the disease. First, those who develop the disease suddenly and die within a few hours, days, or weeks from the beginning. Second, those who begin with slight attacks, provoked only by definite exciting causes, and extending over months or years. The second group is amenable, in a considerable degree, to proper management.

The earliest usual symptom is a mid-sternal or precordial pain, which comes solely with rapid walking, or walking up hill, or ordinary walking against the wind, or on a very cold day. The patient is finally obliged to stand still for a moment or two, upon which the pain disappears, only to return again if the same degree of muscular exertion is resumed. As time passes, particularly if the mode of life is not minutely regulated, the attacks occur as a result of slighter and slighter provocations, such as ordinary walking on the level, the exertion of bathing, brief exposure to cold air, stooping, or a disturbing emotion. Extremely important is the relation of gastrointestinal disturbances to the initiation of an attack. It is very common to observe attacks, which may be serious or fatal, in which gaseous distension of the stomach and intestines, due clearly to avoidable indiscretions in diet, is, without doubt, the exciting cause.

During the early and middle stages of angina pectoris, considered as a disease, the symptoms are largely referable to disturbed innervation and to the sclerotic changes in the arteries. Commonly the pain element is very prominent, and there is usually persistent arterial hypertension. But during these periods the arterial changes progress, and, largely as a result of coronary sclerosis, the myocardium undergoes more or less extensive degenerative changes. When the myocardial disease has increased to an extent sufficient to cause evidences of cardiac insufficiency, the final stage of the disease has begun. Dyspnea, previously slight or absent, appears, and there are physical signs of evanescent or permanent cardiac dilatation. The bases of the lungs are passively congested. There are occasional attacks of general, sometimes acute, pulmonary edema, the liver enlarges, there may be transudates into the serous cavities; in short, the usual witnesses to a failing heart. The painful paroxysms are brief and of slight intensity, and the high arterial tension lessens.

The diagnosis of angina pectoris, at least in its milder forms, cannot be made from the history alone. The other forms of cardiac pain, of toxic or neurotic origin, the latter especially in women, may exactly simulate a true angina pectoris. After allowing due weight to the age, sex, and detailed history of the patient, it is necessary to ascertain the presence or absence of signs of organic disease at the root of the aorta. On this hangs an enormously important decision. In most instances the accessible arteries, the radial, temporal, carotid, femoral, and posttibial, are so palpable or so thickened that a ready diagnosis of general arteriosclerosis can be made. Such widespread changes involve, well-nigh of necessity, the root of the aorta. Possibly, but much more rarely, there may be found the signs of aortic aneurism, aortic insufficiency, or adherent pericardium, with either of which true angina may be associated.

When such plain signs of general arterial or aortic disease co-exists with a history of precordial pain, there need be no hesitation in making a positive diagnosis of true angina pectoris. But it is otherwise in patients with cardiac pain in whom, as may happen, the accessible arteries are soft, and who do not present signs of gross aortic or pericardial lesions. In the election between true and false, organic or functional, there is one physical sign which, I believe, casts the controlling vote. It is so slight, and apparently so insignificant, that one almost hesitates to mention it. It is simply a slight clicking sound, of a harsh or rough quality, accompanying, or following at a barely perceptible interval, the sound of aortic closure. I do not refer to an accentuation of the closure sound of the valve, such as the loud, clean, "cork and bottle" aortic second sound, which is significant of high arterial tension. Nor is it to be confused with a reduplication of the second sound. The slight harsh click, to which I refer, suggests to the ear a definite roughening or thickening of the edges of the aortic cusps. When autopsies have been obtained the findings have justified such an interpretation of this auscultatory sign. If, then, the aortic cusps are roughened, it is certainly a fair inference that the root of the aorta shares in the diseased process.

I recall but two cases of true angina in which this sound has not been heard. It is of course present in many non-anginal cases of aortic disease. But, I wish to repeat, that when one examines a patient with a distinctly anginal history, and yet in whom no evidence of widespread arterial disease can be found, the presence of this sign announces, I believe unmistakably, that the patient has a true stenocardia. Twice I have disregarded the meaning of the presence or absence of this sign. In the first instance the sound was present, but as the patient was comparatively young, only 34 years of age, was not syphilitic, with a blood-pressure of only 100 mm., and had failed to develop anginal pain after vigorous exercise in my presence, I declined to express a definite opinion. A month later he died very suddenly. In the second patient, a man 50 years old, there was a marked general arteriosclerosis, with a history of intermittent breast-pain. Although the pain was not that of a typical angina, inasmuch as its coming was by no means dependent upon muscular exertion, yet it was severe and of notably paroxysmal recurrence. So in spite of the absence of the aortic click-sound, a diagnosis of true angina was made. But one day, about two months later, the patient on entering the room stumbled rather unaccountably. A special examination of the nervous system, which had been neglected, showed absent knee-jerks, incoördination, Argyll-Robertson pupils, and other symptoms in keeping. The supposed angina was simply a higher localisation than is usual of the girdle-pain of locomotor ataxia.

